# Microcystin-LR-Induced Oxidative Stress, Transcriptome Changes, Intestinal Microbiota, and Histopathology in *Rana chensinensis* Tadpoles

**DOI:** 10.3390/ani16020316

**Published:** 2026-01-20

**Authors:** You Wang, Bingjie Wang, Zhuolin He, Jiaxin Chen, Chenyang Liu, Zhanqi Wang, Muhammad Irfan, Lixia Zhang

**Affiliations:** 1 Department of Ecology, College of Life Sciences, Henan Normal University, Xinxiang 453007, China; htu_wangyou@163.com (Y.W.); htu_wbj@163.com (B.W.); htu_hzl@163.com (Z.H.); zhaojinxin122000@163.com (J.C.); htu_lcy@163.com (C.L.); 2 Key Laboratory of Vector Biology and Pathogen Control of Zhejiang Province, College of Life Sciences, Huzhou University, Huzhou 313000, China; zhqwang@zju.edu.cn; 3 Department of Biotechnology, University of Sargodha, Sargodha 40100, Pakistan; irfan.ashraf@uos.edu.pk; 4 Puyang Field Scientific Observation and Research Station for Yellow River Wetland Ecosystem, Puyang 457183, China

**Keywords:** MC-LR, *Rana chensinensis* tadpoles, oxidative stress, liver transcriptome, intestinal microbiota, histopathology

## Abstract

Microcystin-LR (MC-LR), produced by Cyanobacteria, is being detected in many types of waters, posing a universal threat to aquatic animals. However, there have been few comprehensive endpoints assessed, including oxidative stress, transcriptome changes, intestinal microbiota, and histopathology, in anurans exposed to MC-LR. In this study, all these effects of MC-LR on Chinese brown frog (*Rana chensinensis* David, 1875) tadpoles were investigated by exposing the tadpoles to MC-LR at different concentrations (0, 0.1, 1.0, 5.0, and 10.0 μg/L) for 7 days. Our results revealed that treatment of tadpoles with the high MC-LR dosage (10.0 μg/L) induced a significant increase in malondialdehyde (MDA) content and decreases in superoxide dismutase (SOD) activity, glutathione peroxidase (GPx) activity, and total antioxidant capacity (TAC). RNA-seq analysis of the liver showed that the number of differentially expressed genes (DEGs) was 2361 under lower MC-LR stress (1.0 μg/L), while the number of DEGs increased to 3185 under higher MC-LR stress (10.0 μg/L). Gene Ontology analysis showed that several biological processes and molecular functions related to digestion were enriched in both MC-LR treated groups, such as digestion, serine-type endopeptidase activity, and serine-type peptidase activity. KEGG enrichment analysis also indicated that the digestion for pancreatic secretion, protein digestion and absorption, and fat digestion and absorption pathways were significantly enriched in the treatment groups. Additionally, the bacterial richness was elevated by MC-LR exposure. At the phylum level, treatment with MC-LR changed the relative abundances of Desulfobacterota, Fusobacteriota, and Actinobacteriota. At the genus level, MC-LR caused significant alterations in the abundances of 23 genera. Furthermore, examination of sections obtained from the livers and intestines of tadpoles in the treatment groups showed damaged histological structure. The knowledge from this study will have potential value for understanding the mechanisms related to MC-LR toxicity in anurans.

## Simple Summary

Microcystin-LR (MC-LR), a toxic byproduct of harmful algal blooms, has emerged as a global health threat to aquatic organisms. However, its toxic effects on amphibian tadpoles remain incompletely understood. This study presents a comprehensive analysis of MC-LR-induced toxicity in the liver and intestine of Chinese brown frog (*Rana chensinensis* David, 1875) tadpoles by integrating assessments of oxidative stress, liver transcriptome, gut microbiota, and histopathology. Our results demonstrated that exposure to MC-LR induced oxidative stress and downregulated genes involved in digestion in tadpole livers. The richness of the intestinal microbiota and the abundances of the dominant taxa were altered under MC-LR stress. Further, MC-LR caused structural damage in both the liver and intestine. These findings will expand our understanding of the toxic mechanisms induced by MC-LR in anuran tadpoles.

## 1. Introduction

The past few decades have witnessed a significant increase in nutrient loading, particularly nitrogen and phosphorus, into aquatic ecosystems, thereby leading to frequent outbreaks of cyanobacterial blooms [1,2]. Microcystins (MCs), highly toxic secondary metabolites released by Cyanobacteria during harmful algal blooms, are becoming a major environmental problem worldwide [3,4]. Previous studies have reported their bioaccumulation in fish [5], shrimp [6], and crabs [7], which could decrease their growth rate and cause injury to multiple organs and tissues, including the liver, intestine, pancreas, and gonads [4,8]. Microcystin-LR (MC-LR) is acknowledged as one of the most prevalent and hazardous isomers of MCs, posing a great threat to aquatic species [9,10,11]. Therefore, many toxicological studies have been conducted to clarify the harmful effects of MC-LR on aquatic animals.

A key mechanism of MC-LR toxicity involves the occurrence of oxidative stress. This stress arises from a disturbed redox homeostasis, leading to reactive oxygen species (ROS) overproduction and antioxidant defense dysregulation [12,13,14,15,16]. For example, the results of a study on common carp (*Cyprinus carpio* Linnaeus, 1758) demonstrated that MC-LR induced oxidative damage in the liver, as evidenced by markedly enhanced glutathione S-transferase (GST) activity, decreased glutathione (GSH) content, and increased malondialdehyde (MDA) level [17]. After MC-LR injection at a concentration of 29.30 μg/kg, Chinese mitten crab (*Eriocheir sinensis* H. Milne Edwards, 1853) exhibited reduced catalase (CAT) and glutathione peroxidase (GPx) activities at 6 h post-injection, and a significant rise in MDA content was observed after 12 h [18]. In the presence of MC-LR, the production of ROS and MDA increased, accompanied by decreases in superoxide dismutase (SOD) activity and the total antioxidant capacity (TAC), in zebrafish (*Danio rerio* Hamilton, 1822) embryos [19]. In addition, it was found that MC-LR accumulated in the hepatopancreas of crabs (*Neohelice granulate* Dana, 1851) and induced oxidative damage [20]. All of these results revealed that MC-LR exposure could disrupt the balance of the antioxidant defense system in aquatic animals.

As a hepatotoxic agent, MC-LR will target the liver primarily and lead to liver bleeding, or even necrosis [21]. The livers of zebrafish exposed chronically to MC-LR for 90 days showed structural damage, mitochondrial dysfunction, and lipid metabolic disturbance [1]. Additionally, a range of pathological alterations in the liver of common carp appeared after exposure to MC-LR for 14 days [14]. At the gene level, Chi et al. [7] found differentially expressed genes (DEGs) related to MC-LR stress in Chinese mitten crab samples by transcriptome sequencing, and these DEGs were mainly involved in lipid metabolism, apoptosis, and immunization. Meanwhile, immune- and redox-related gene expression levels in the hepatopancreatic transcriptome in crayfish (*Procambarus clarkii* Charles Girard, 1852) were significantly changed by MC-LR [22]. Moreover, structural damage in the liver of Nile tilapia (*Oreochromis niloticus* Linnaeus, 1758) had a concentration-dependent relationship with MC-LR, and transcriptomic analysis revealed that the DEGs were associated with oxidative stress, apoptosis, and metabolism [23]. Therefore, the use of RNA sequencing analysis combined with histopathology shows the potential to elucidate the mechanisms of MC-LR-induced hepatotoxicity.

MC-LR can be transported into and accumulate in intestinal epithelial cells, ultimately inducing severe histopathological alterations [24,25,26,27,28]. There was obvious damage to intestinal histology in crayfish [29] and Pacific white shrimp (*Litopenaeus vannamei* Boone, 1931) [30] exposed to MC-LR. As we know, the intestinal microbiota provides essential nutrients for the hosts [31,32], is involved in metabolism [33], and plays a vital role in immune regulation [34]. Normal intestinal function is based on a stable and balanced microbiota [35]. However, the diversity and composition of gut microbial communities could be altered by MC-LR exposure. Concretely, MC-LR exposure changed microbial diversity and disturbed microbial composition in Pacific white shrimp [30]. Moreover, significant variations in the relative abundances of several bacterial taxa were found when comparing the experimental and control groups [30]. Similar results were shown in zebrafish: MC-LR induced significant changes in both the abundance and the diversity of the intestinal core microbiota, consequently elevating susceptibility to pathogenic colonization [36,37]. As for young African clawed toads (*Xenopus laevis* Daudin, 1802), the severity of intestinal injury exhibited a concentration-dependent response to MC-LR exposure, and their gut microbiota composition varied significantly at different MC-LR concentrations [38]. In summary, MC-LR exposure will induce histopathological damage to the intestine and affect the diversity and composition of the gut microbiota.

The Chinese brown frog (*Rana chensinensis* David, 1875) is a distinctive amphibian species that is distributed widely in northern China [39]. It is reported that Chinese brown frog tadpoles are extremely sensitive to aquatic pollution because of their completely aquatic lifestyle and highly permeable skin and gills, making them a vital indicator for monitoring the adverse effects of toxic contaminants in natural water bodies [40,41,42]. Exposure to environmental pollutants has been shown to elicit a spectrum of harmful effects in Chinese brown frog tadpoles, including reduced histological indices, gut microbial dysbiosis, perturbed lipid metabolism, and impaired life-history traits [41,42,43]. MC-LR is a common water pollutant with high toxicity, the adverse impacts of which on amphibian larvae have been extensively documented [44,45,46,47,48,49,50]. However, its toxic effects on Chinese brown frog tadpoles have not been determined. To bridge this knowledge gap, this study conducts a comprehensive assessment of MC-LR toxicity in Chinese brown frog tadpoles by integrating oxidative stress, liver transcriptome, gut microbiota, and histology data. The aim is to perform a comprehensive evaluation of the health risk that MC-LR poses to Chinese brown frog tadpoles and to provide deep insights into the adverse impacts of MC-LR on anurans.

## 2. Materials and Methods

### 2.1. Chemicals and Reagents

MC-LR (purity ≥ 95%) was purchased from Sigma-Aldrich Co., Ltd. (St. Louis, MO, USA). MS-222 (ethyl 3-aminobenzoate methanesulfonate, 98%) was bought from Shanghai Macklin Biochemical Co., Ltd. (Shanghai, China). The hematoxylin and eosin (H&E) staining solution, paraformaldehyde (4%), and phosphate-buffered saline (PBS) (1×, pH 7.2–7.4, 0.01 M) were obtained from Beijing Solarbio Science and Technology Co., Ltd. (Beijing, China). Ethanol (75%) was supplied by Xinxiang Xianfeng Medical New Material Co., Ltd. (Xinxiang, China). Commercial assay kits for the detection of oxidative stress markers were acquired from Beyotime Biotechnology Co., Ltd. (Shanghai, China).

### 2.2. Animals and Samples

Chinese brown frog tadpoles with an initial body length of 40.72 ± 3.17 mm and body weight of 786.52 ± 112.39 mg were collected from Tianjie Mountain, Henan Province, China (113°36′0″ E, 35°36′36″ N) during April and May in 2022. We identified the developmental stages of tadpoles following the developmental staging table of Gosner [51]. Tadpoles at Gosner stage (Gs) 28 were selected for the experiment, because the tadpoles at this stage displayed frequent foraging activities in water [52]. Before the formal experiments, a total of 300 tadpoles were divided into 15 independent open-top polycarbonate tanks, each of which had an effective volume of 4 L. The temperature was maintained at 17 ± 1 °C using a dedicated heater (CS-60, Zhongshan Xiaolan JBA Electrical Appliance Co., Ltd., Zhongshan, China). In addition, key water quality parameters, including pH, dissolved oxygen, ammonia nitrogen, nitrite, sulfate, and total hardness, were maintained at 7.0 ± 0.5, 6.60 ± 0.1 mg/L, 0.3 mg/L, 0.3 mg/L, 25 mg/L, and 160 mg/L, respectively. We reared tadpoles on 1% of the total biomass once daily using commercial feed for an acclimation period of 7 days. After acclimation, the tadpoles reached Gs 30. Subsequently, each of the 15 tanks was randomly assigned to an MC-LR treatment group or a control group. There were 60 tadpoles in each treatment group, and each replicate group had 20 tadpoles. Three biological replicates (n = 3) were performed for each exposure level.

The permissible limit of MC-LR in drinking water, as permitted by the WHO, is 1.0 μg/L [53]. In eutrophic natural waters, the concentration of MC-LR typically ranges from 0.1 to 10.0 μg/L [17]. To comprehensively estimate MC-LR toxicity to Chinese brown frog tadpoles, five MC-LR concentration gradients were set up, as follows: 0, 0.1, 1.0, 5.0, and 10.0 μg/L. One-third of the dechlorinated water in tanks was replaced with water containing identical MC-LR concentrations every day to maintain stable MC-LR levels throughout the experimental period [54,55]. The feeding regime during exposure was same as that during the acclimation phase. Food residues were removed daily from each tank, indicating sufficient food supply. All tadpoles survived to the end of the experiment.

Ten tadpoles were selected randomly from each replicate tank after MC-LR exposure for 7 days. This period is considered ecologically relevant to the lifetime of tadpoles [52]. Next, we obtained the livers from the tadpoles after anesthesia with 300 mg/L buffered MS-222. Due to limited tissue available from individual tadpoles, the livers of five tadpoles in each replicate group were mixed with refrigerated PBS solution in a ratio of 1:4 (*w*/*v*) and ground with a frozen mixed ball mill (MM 400; RETSCH, Haan, Germany). Then, the prepared samples were separated in a refrigerated centrifuge (Sigma 3K15, SIGMA, Darmstadt, Germany) at 12,000 rpm for 5 min at 4 °C. The supernatants were obtained to measure oxidative stress markers, and the precipitate was cryopreserved in liquid nitrogen for transcriptomic analysis.

Subsequently, we sacrificed another five tadpoles from each replicate tank. Following dissection, the liver and gut samples were immersed in 4% paraformaldehyde fixative solution to evaluate histological damage caused by MC-LR. In addition, the gut contents from five individuals within the same tank were obtained, mixed, and frozen immediately in liquid nitrogen for intestinal microbiota analysis. To avoid cross-infection, we washed all instruments with 75% ethanol and used new disposable gloves when dissecting each tadpole. All experimental procedures were performed under sterile conditions on a super clean bench (SW-CJ-2FD, Suzhou Purifying Equipment Co., Ltd., Suzhou, China).

### 2.3. Measurement of Oxidative Stress Markers

MDA content, SOD activity, GPx activity, and TAC in liver samples were analyzed with commercial kits. Briefly, MDA content was quantified by spectrophotometric analysis at 535 nm of the red MDA-TBA adduct formed through the reaction between MDA and 2-thiobarbituric acid (TBA) [56]. The superoxide anion radical (O^2−^) produced from xanthine in the presence of xanthine oxidase can reduce nitroblue tetrazolium (NBT) to formazan. The photochemical reduction of NBT at 560 nm is inhibited by SOD, which was used as the basis for assays of SOD activity [57]. The activity of GPx was measured at a wavelength of 340 nm by recording the nicotinamide adenine dinucleotide phosphate (NADPH) concentration decrease [58]. TAC, the sum of all antioxidants, was measured using the 2,2′-azino-bis (3-ethylbenzthiazoline-6-sulfonic acid) (ABTS) method, which utilized quenching of ABTS radical cations by antioxidants. ABTS radical cations are generated through peroxidase oxidation, and the decrease in its absorbance at 414 nm induced by antioxidants was quantified [59]. All assays were conducted with a microplate reader (SH1M2F, Agilent, Santa Clara, CA, USA). Using SPSS software (version 22.0), statistical differences in these markers among the groups were assessed using one-way analysis of variance (ANOVA) with Tukey’s post hoc test.

### 2.4. RNA Isolation, Library Preparation, and Transcriptome Sequencing

Invitrogen TRIzol reagent (Ambion, Austin, TX, USA) was employed for extraction of total RNA from the liver samples. The RNA integrity was then assessed using an RNA Nano 6000 assay kit and a Bioanalyzer 2100 system (Agilent Technologies, Santa Clara, CA, USA). A cDNA library was constructed, and 150-bp paired-end sequencing was performed on an Illumina X PLUS platform (Illumina, San Diego, CA, USA) with a sequencing data volume of 6G. More details are presented in Supplementary Method S1.

### 2.5. Transcriptome Assembly and DEG Screening

Raw sequencing data were transformed to Fastq format (raw data). High-quality clean reads were generated from the raw RNA-Seq data by filtering out adapter sequences, low-quality sequences, poly-N sequences, and other low-quality reads (Q-values < 20) using Fastp (version 0.22.0) with default parameters. Subsequently, we performed quality control of the filtered reads by calculating sequence duplication level, Q20, Q30, and GC content. The Trinity program (version 2.5.1) was used to de novo assemble transcripts, and all parameters were set to default. Gene expression levels were quantified by FPKM values using RSEM (version 2.15, default parameters) for differential expression analysis. Thresholds of |log2FoldChange| > 1 and *p* < 0.05 were considered as criteria to identify DEGs using DESeq software (version 1.32.0). Subsequently, GO enrichment analysis was carried out using topGO (version 2.32.0) with default settings to identify the significantly enriched biological processes of DEGs. Finally, we performed KEGG enrichment analysis to assess the significant signaling pathways of DEGs with ClusterProfiler (version 4.6.0), using default parameters. Significantly enriched terms and pathways were screened using a cutoff of a false discovery rate (FDR) < 0.01.

### 2.6. Microbial DNA Extraction and PCR Amplification

Total microbial genomic DNA from intestinal contents was extracted using a Soil DNA Extraction Kit (Shanghai MeiJiYuHua Co., Ltd., Shanghai, China), following the manufacturer’s instruction. Subsequently, the hypervariable V3-V4 region of bacterial 16S rRNA was targeted for amplification using universal primers 338F/806R [60]. The details of the PCR amplification are provided in Supplementary Method S2.

### 2.7. Illumina Miseq Sequence Processing

Following purification with an AxyPrep DNA Gel Extraction Kit (Axygen Biosciences, Union City, CA, USA), the PCR amplicons were quantified using a QuantiFluor^TM^-ST system (Promega, Madison, WI, USA) and pooled at equimolar ratios based on the concentration of each amplicon. Next, the resulting PCR products were sequenced using an Illumina MiSeq platform, with the detailed methodology described in Supplementary Method S3.

### 2.8. Biodiversity Analysis

The gut microbial diversity and composition of tadpoles in all groups were assessed via 16S rRNA gene sequencing. Alpha diversity indices (Chao, Ace, Shannon, and Simpson indices) were calculated using MOTHUR (version 1.30.2) to evaluate the microbial community richness and diversity for each sample. Rarefaction curves were used as a qualitative method to estimate sequencing depth and were obtained using USEARCH (version 7). For beta diversity analysis, principal coordinate analysis (PCoA) plots based upon Bray-Curtis distance were created to visualize bacterial community differences among groups, with all analyses conducted in R (version 3.3.1). The PERMANOVA test was applied to test differences in the microbial profiles. Also, QIIME software (version 1.9.1) was used to analyze bacterial abundance and composition. Relative abundance histograms were generated using Origin software (version 2016). The statistical data were analyzed by SPSS software using one-way ANOVA and Tukey’s test. All results are expressed as mean ± standard deviation (SD), and the significance level was set as *p* < 0.05.

### 2.9. Histopathology Examination

The experimental procedure was conducted according to a prior study [61]. After fixation, all liver and intestine samples were further dehydrated with ethanol series of 50% to 95% and cleared with xylene. Next, the tissues were embedded in paraffin and then sliced to a thickness of 4 μm using a microtome (RM2245, Leica, Wetzlar, Germany). Hematoxylin and eosin stain was used for microscopic examination of the sectioned tissues. The histological sections were examined under a fluorescence microscope (Olympus BX63, Tokyo, Japan) to observe histological changes.

## 3. Results

### 3.1. Effect of MC-LR Exposure on Oxidative Stress Markers in Livers

**Figure 1:**
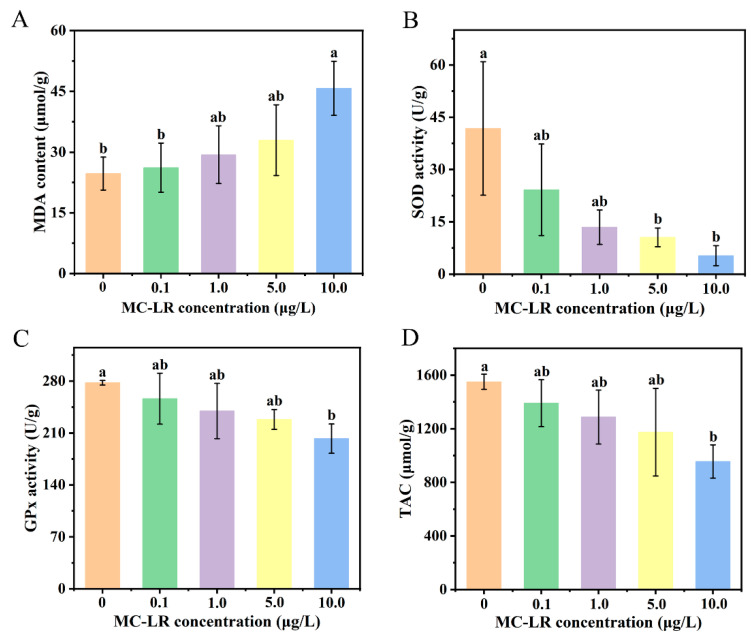
Effects of MC-LR exposure on oxidative stress markers in the livers of Chinese brown frog tadpoles. (**A**) MDA content; (**B**) SOD activity; (**C**) GPx activity; (**D**) TAC. Values are presented as the mean ± SD. Letters (a and b) indicate significant differences (*p* < 0.05) among the groups.

The MDA content exhibited a dose-dependent increase, with significant elevations observed at 10.0 μg/L MC-LR compared to both the control and 0.1 μg/L treatment groups (Figure 1A, both *p* < 0.05). SOD activity was significantly suppressed after MC-LR exposure at 5.0 and 10.0 μg/L (Figure 1B, both *p* < 0.05). Furthermore, treatment of the tadpoles with 10.0 μg/L MC-LR induced statistical decreases in the GPx activity and TAC relative to the control group (Figure 1C,D, both *p* < 0.05).

### 3.2. Liver Transcriptome Sequencing of Chinese Brown Frog Tadpoles in Response to MC-LR Exposure

To develop the appropriate MC-LR exposure regimen using effective concentrations, we selected two MC-LR concentrations, 1.0 μg/L (ATr) and 10.0 μg/L (BTr), to perform liver transcriptome and gut microbiota analysis. The transcriptome sequencing results of Chinese brown frog tadpole livers were presented in Table S1. We obtained between 47,595,232 and 54,152,640 clean reads across all samples, with Q20 and Q30 scores exceeding 98.67% and 96.54%, respectively. In total, the de novo sequence assembly of clean reads produced 130,648 unigenes with an average length of 887 bp and an N50 score of 1178 bp.

### 3.3. Identifying DEGs in Chinese Brown Frog Tadpoles Exposed to MC-LR

There were 2361 DEGs in the ATr group compared with the Con group, including 722 upregulated genes and 1639 downregulated genes (Figure S1A). A total of 3185 DEGs were identified in the BTr group, of which 840 genes were upregulated and 2345 genes were downregulated (Figure S1B). Furthermore, five key DEGs were identified in all samples. Their expression profiles in relation to MC-LR exposure levels were visualized in a heatmap (Figure S2). Among them, four genes (*CELA2A*, *Tff2*, *CELA1*, and *PNLIP*) were downregulated in exposed liver tissue, while *FCN2* expression was upregulated.

**Figure 2:**
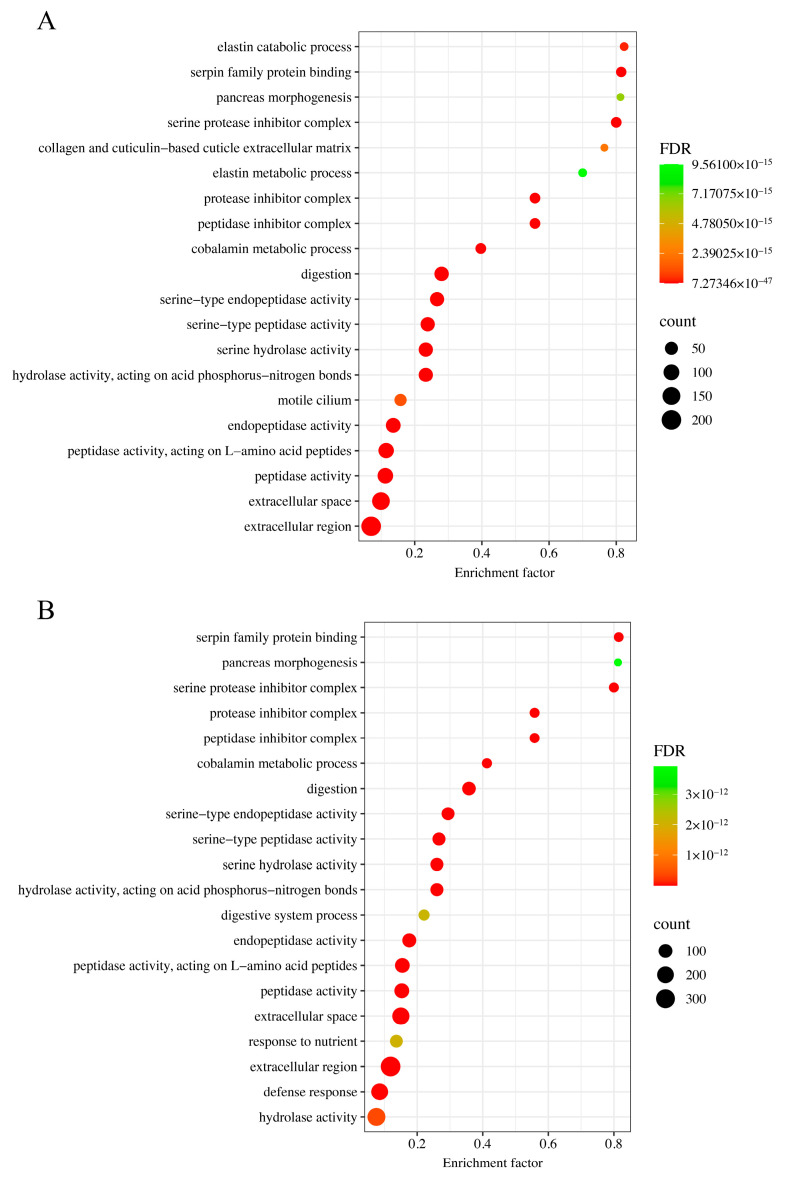
GO enrichment analysis of differentially expressed genes (DEGs) in the livers of Chinese brown frog tadpoles exposed to MC-LR. (**A**) The top 20 enriched GO terms in the 1.0 μg/L MC-LR-treated group; (**B**) the top 20 enriched GO terms in the 10.0 μg/L MC-LR-treated group.

To identify the biological functions and signaling pathways implicated in the transcriptomic changes, GO and KEGG enrichment analyses were subsequently conducted. With an FDR cutoff of 0.01, MC-LR exposure at concentrations of 1.0 μg/L (ATr) and 10.0 μg/L (BTr) resulted in significant enrichment of 148 and 484 GO terms, respectively. Given the large number of significantly enriched GO terms, we focused on the top 20 most significantly enriched terms in each comparison (ATr vs. Con and BTr vs. Con), as illustrated in Figure 2. Compared to the Con group, ‘digestion’ was the most significantly enriched GO term in ATr group, followed by ‘serine-type endopeptidase activity’, ‘serine-type peptidase activity’, ‘hydrolase activity, acting on acid phosphorus-nitrogen bonds’, ‘serine hydrolase activity’, ‘extracellular space’, and ‘extracellular region’ (Figure 2A). In the BTr group, ‘extracellular region’, ‘digestion’, ‘extracellular space’, ‘serine-type endopeptidase activity’, ‘serine-type peptidase activity’, and ‘hydrolase activity, acting on acid phosphorus-nitrogen bonds’ were the most significantly enriched GO terms (Figure 2B). The details about the top 20 enriched GO terms in ATr and BTr groups were displayed in Tables S2 and S3, respectively. It was worth mentioning that the significantly enriched GO terms ranked most highly in both treatment groups were ‘digestion’, ‘serine-type endopeptidase activity’, ‘serine-type peptidase activity’, ‘extracellular space’, and ‘extracellular region’.

**Figure 3:**
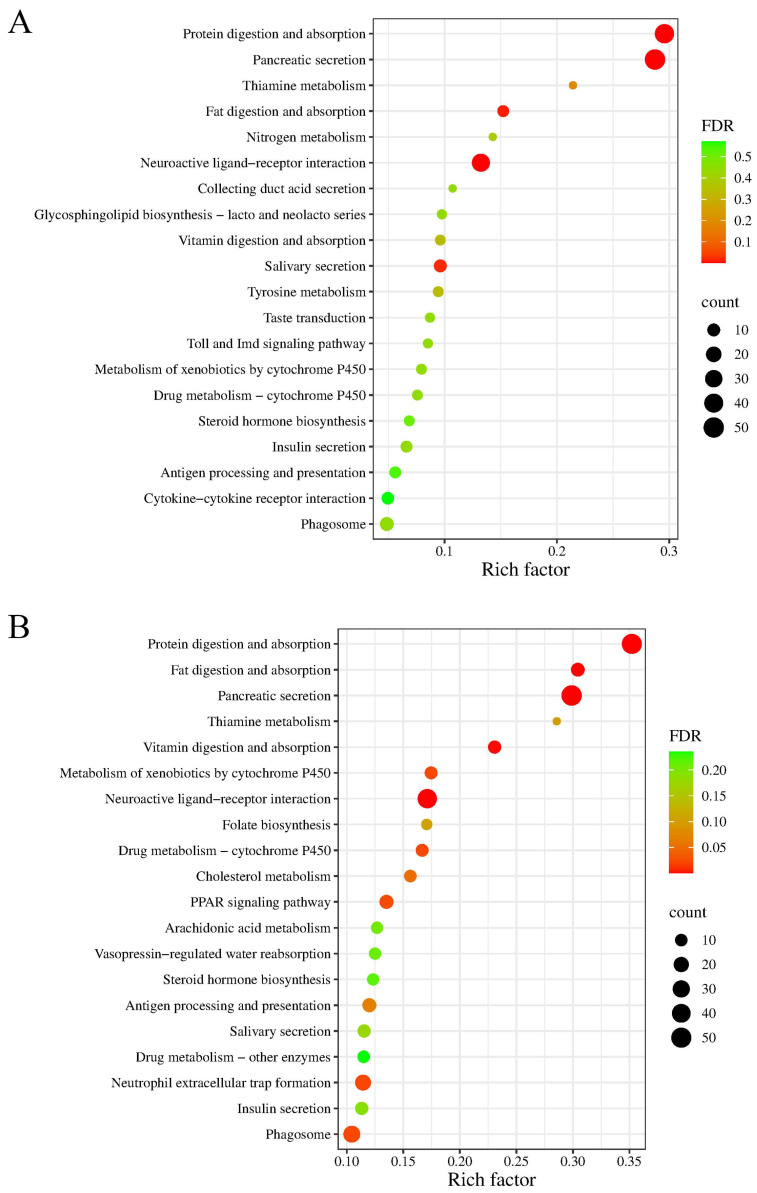
KEGG enrichment analysis of differentially expressed genes (DEGs) in the livers of Chinese brown frog tadpoles exposed to MC-LR. (**A**) The top 20 enriched KEGG pathways in the 1.0 μg/L MC-LR-treated group; (**B**) the top 20 enriched KEGG pathways in the 10.0 μg/L MC-LR-treated group.

Furthermore, DEGs were functionally annotated using the KEGG database to reveal key biological pathways modulated by MC-LR in tadpoles. Figure 3 displayed the top 20 enriched KEGG pathways in the MC-LR-exposed groups. In detail, we found 5 and 11 significantly enriched KEGG pathways in the ATr group and the BTr group (FDR < 0.01), respectively. Among these pathways, the digestive system (namely ‘pancreatic secretion’, ‘protein digestion and absorption’, and ‘fat digestion and absorption’) accounted for the greatest number of DEGs in two treatment groups. Tables S4 and S5 showed the detailed information of significantly enriched KEGG pathways in the ATr group and the BTr group, respectively. Overall, the enrichment patterns observed in both GO terms and KEGG pathways implicated the response mechanisms of Chinese brown frog tadpoles to MC-LR exposure.

### 3.4. Summary of the 16S rRNA Sequencing Data

A total of 460,771 high-quality sequences were generated from the gut samples of tadpoles, which were grouped into 1229 operational taxonomic units (OTUs) at a 97% identity threshold. The alpha diversity rarefaction curves indicated that the sequencing depth sufficiently captured the biodiversity in all samples (Figure S3).

### 3.5. Intestinal Microbial Diversity

Chao, Ace, Shannon, and Simpson indices were used to evaluate the alpha diversity of the intestinal microbial communities. As shown in Table 1, the Chao and Ace indices in both experimental groups (ATr and BTr) were higher than those in the control (Con) group. Significant relationships were observed between the ATr group and the Con group (both *p* < 0.05), whereas the BTr group showed no statistical variation compared to the control group (both *p* > 0.05). In addition, PCoA analysis demonstrated a significant difference in microbial community structure among the three groups (PERMANOVA, R^2^ = 0.603, *p* = 0.017, Figure S4).

### 3.6. OTU Distribution

The distribution of OTUs was visualized using Venn diagrams to enumerate the numbers of group-specific and shared OTUs (Figure S5). There were 214 OTUs that existed only in Con group, indicating that these OTUs were susceptible to MC-LR. In addition, the numbers of unique OTUs identified in the ATr and BTr groups were 268 and 260, respectively. Those OTUs likely represent resistant taxa within the gut microbiome. Moreover, 255 OTUs were shared among all three groups.

### 3.7. Intestinal Microbial Composition

**Figure 4:**
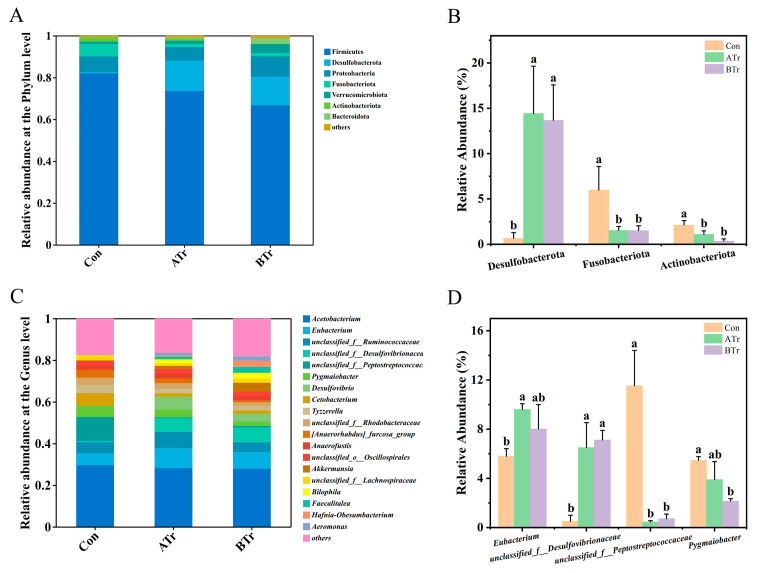
Effects of MC-LR exposure on the gut microbiota of Chinese brown frog tadpoles. (**A**) Relative abundances of dominant phyla; (**B**) phyla with significant variations in relative abundance after MC-LR exposure; (**C**) relative abundances of dominant genera; (**D**) genera with significant variations in relative abundance after MC-LR exposure. Letters (a and b) indicate significant differences (*p* < 0.05) among the groups. Con: 0 μg/L MC-LR; ATr: 1.0 μg/L MC-LR; BTr: 10.0 μg/L MC-LR.

At the phylum level, Firmicutes (53.79–86.64%), Desulfobacterota (0.25–20.44%), Proteobacteria (5.79–12.54%), and Fusobacteriota (0.99–8.20%) were the dominant phyla in Chinese brown frog tadpoles (Figure 4A). Compared with the Con group, the abundance of Desulfobacterota was significantly increased in both treatment groups, while the relative abundance of Fusobacteriota decreased significantly (Figure 4B). We also observed reduced Actinobacteriota abundance after MC-LR exposure (Figure 4B). Furthermore, the Firmicutes/Bacteroidetes ratio was lower in both MC-LR-treated groups than that in the Con group (ATr vs. Con = 0.45:1; BTr vs. Con = 0.19:1). At the genus level, Chinese brown frog tadpoles were enriched in *Acetobacterium* (18.73–36.63%), *Eubacterium* (5.06–10.13%), *unclassified_f__Ruminococcaceae* (3.58–9.61%), *unclassified_f__Desulfovibrionaceae* (0.20–8.54%), and *unclassified_f__Peptostreptococcaceae* (0.34–13.61%) (Figure 4C). In total, 23 genera (relative abundances > 1%) showed significant differences in relative abundance after exposure to MC-LR. Given the large number of genera with significant variations, only the top four taxa were presented in Figure 4D. MC-LR exposure significantly increased the relative abundance of *Eubacterium*, while decreasing that of *Pygmaiobacter,* compared with the control specimens. Furthermore, the abundances of two unclassified genera had different responses to MC-LR exposure.

### 3.8. Effects of MC-LR Exposure on Liver and Intestinal Histology

**Figure 5:**
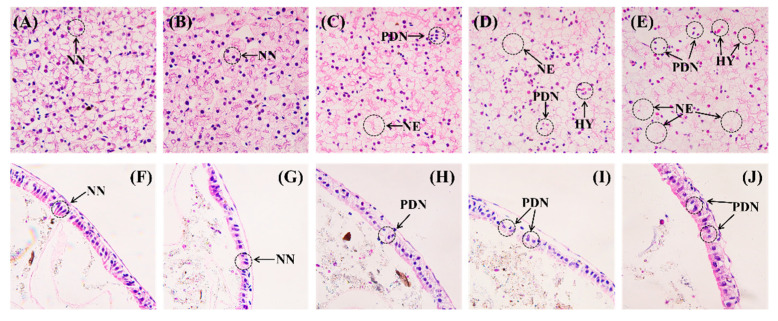
Light micrographs of the liver and intestinal histology of Chinese brown frog tadpoles exposed to MC-LR. Representative micrographs of liver sections collected from the (**A**) control group; (**B**) 0.1 μg/L MC-LR-treated group; (**C**) 1.0 μg/L MC-LR-treated group; (**D**) 5.0 μg/L MC-LR-treated group; and (**E**) 10.0 μg/L MC-LR-treated group. Representative micrographs of intestine sections collected from the (**F**) control group; (**G**) 0.1 μg/L MC-LR-treated group; (**H**) 1.0 μg/L MC-LR-treated group; (**I**) 5.0 μg/L MC-LR-treated group; and (**J**) 10.0 μg/L MC-LR-treated group. NN: normal nucleus; NE: necrosis; PDN: pyknotic and deformed nuclei; HY: hyperemia. H&E stain, 40×.

Tadpoles in the control group exhibited normal liver structures, in which the hepatocytes were distributed uniformly (Figure 5A). No significant histological changes were found in the livers of tadpoles exposed to 0.1 μg/L MC-LR (Figure 5B). Minor necrosis occurred in the 1.0 μg/L MC-LR-treated group, and a small proportion of nuclei exhibited pyknosis and deformation (Figure 5C). In contrast, exposure to 5.0 and 10.0 μg/L MC-LR induced severe hepatic injury, which was characterized by extensive necrosis, numerous pyknotic and deformed nuclei, and slight hyperemia (Figure 5D,E). Histopathological assessment using a scoring system for hepatic lesions revealed that a score of 3 was assigned to liver tissues in tadpoles from the high-dose treatment groups (Table S6).

The intestinal histology of tadpoles was investigated using H&E staining. The results demonstrated that the tadpoles without MC-LR exposure had intact intestinal structures, characterized by regular mucosal epithelial cells, normal nuclear shape, and clear cell boundaries (Figure 5F). There were no obvious changes in the intestinal morphology of tadpoles exposed to 0.1 μg/L MC-LR (Figure 5G). In the 1.0 μg/L MC-LR treatment group, some epithelial cells had pyknotic and deformed nuclei (Figure 5H). However, severe histological alterations, including disordered epithelial cells, indistinct cell boundaries, and numerous pyknotic and deformed nuclei were observed in samples from the high-dose groups (Figure 5I,J).

## 4. Discussion

While many studies have been devoted to elucidating the toxicity of MC-LR to amphibians, a comprehensive investigation into its specific effects on Chinese brown frog tadpoles has not been performed. In the present study, we examined the effects of MC-LR exposure on Chinese brown frog tadpoles by assessing alterations in oxidative stress, liver transcriptome, intestinal microbiota, and histopathology. The results indicated that exposure to high-dose MC-LR induced significant variations in liver oxidative stress markers as compared to control tadpoles, including an increase in MDA content and decreases in SOD activity, GPx activity, and TAC. GO and KEGG enrichment analyses revealed that a significant proportion of DEGs was associated with digestion-related pathways. Furthermore, MC-LR exposure changed the richness and composition of the gut microbiota and induced histopathological damages in both liver and intestine tissues. All these findings elucidated harmful impacts of MC-LR exposure on the liver and intestine of Chinese brown frog tadpoles.

As the fundamental mechanism of MC-LR toxicity, oxidative stress occurs with overproduction of ROS that disrupts redox homeostasis [62,63]. MDA is the end-product of lipid peroxidation, which has been extensively validated as a reliable indicator of oxidative stress [64,65]. Zhang et al. [66] found that the levels of MDA always showed increases in fish and mammals exposed to MC-LR on the basis of data from 67 studies. Furthermore, a marked increase in MDA level was also found in the hepatopancreas of Chinese mitten crabs following 12 h of exposure to MC-LR [67]. In this study, MDA content exhibited a concentration-dependent increase in response to MC-LR, and statistically significant elevations were observed at 10.0 μg/L MC-LR compared to both the control and 0.1 μg/L treatment groups. The results indicated that MC-LR exposure promoted lipid peroxidation in tadpole livers. Correspondingly, the antioxidant defense system was disrupted by MC-LR. SOD is capable of converting the superoxide radical into the less reactive H_2_O_2_ [68]. Our results showed that SOD activity decreased with increasing MC-LR dosage, and significant differences were found between the high-dose treatment groups (5.0 and 10.0 μg/L) and the control group. This decline was consistent with changes in exposed bighead carp (*Hypophthalmythys nobilis* Richardson, 1845) larvae [69] and Tenca fish (*Tinca tinca* Linnaeus, 1758) [70]. The observed reduction in SOD activity suggested that MC-LR exposure may suppress enzymatic antioxidant defense systems, potentially through excessive production of superoxide radicals [60,71]. GPx is a critical enzyme for the reduction of lipid hydroperoxides and H_2_O_2_, which usually works in concert with GSH in MC-LR detoxification and protection of peripheral tissues such as the liver [72,73]. In Chinese brown frog tadpoles, the activity of GPx was significantly suppressed by 10.0 μg/L MC-LR treatment, similar to the black-spotted frog (*Rana nigromaculata* Hallowell, 1860) [16], zebrafish [74], and Chinese mitten crabs [75]. This depression in hepatic GPx activity showed a failure of antioxidant defenses under elevated oxidative stress, ultimately resulting in hepatic injury. TAC is used to assess the integrated antioxidant capacity of cells [76]. In the present study, the level of TAC decreased as MC-LR concentration increased, and it was significantly lower in the 10.0 μg/L MC-LR-treated group than in the control group. This trend was consistent with what has been found previously in zebrafish [19,77] and Chinese mitten crabs [67], indicating that the antioxidant capacity of tadpole livers declined after MC-LR treatment. Taken together, the increased MDA and the decreases in SOD, GPx, and TAC offered comprehensive evidence that exposure to MC-LR triggered a state of oxidative stress by reducing the antioxidant defense system in tadpoles. This compromised antioxidant capacity subsequently failed to scavenge ROS, resulting in the accumulation of ROS in cells [12]. Excessive ROS could target multiple biomolecules, such as DNA, RNA, proteins, lipids, and enzymatic cofactors, which would cause various cellular responses, including DNA damage, protein oxidation and inactivation, lipid peroxidation, and enzyme activity variations, ultimately disrupt normal cellular metabolism [78,79]. Correspondingly, the expression of genes essential for maintaining normal physiological processes will be altered.

RNA sequencing has become an indispensable tool for transcriptome analysis and was used to conduct a comprehensive analysis of differential gene expression in tadpole livers exposed to MC-LR [80]. In MC-LR-exposed tadpoles, the most significantly enriched GO terms (e.g., ‘digestion’, ‘serine-type endopeptidase activity’, ‘serine-type peptidase activity’, and ‘hydrolase activity, acting on acid phosphorus-nitrogen bonds’) and KEGG pathways (e.g., ‘pancreatic secretion’, ‘protein digestion and absorption’, and ‘fat digestion and absorption’) were primarily associated with the digestive system. Notably, most of the DEGs involved in these functional categories were downregulated. It was reported that the digestive system exhibited high susceptibility to ROS [81]. Both *CELA1* and *PNLIP* are predominant digestive enzymes [82,83]. Their transcript levels were decreased in exposed tadpoles. In addition, digestive enzyme activity is subject to modulation by external factors capable of influencing metabolic function [81,84,85]. In this study, we observed widespread downregulation of DEGs associated with digestive enzyme activity in Gene Ontology analyses, indicating the inhibition of crucial enzymes involved in the digestive process. The pancreas is essential for nutrient metabolism, because the exocrine cells in the pancreas can secrete digestive enzymes [86,87]. *CELA2A* plays a key role in pancreatic secretion [88]. The downregulation of the *CELA2A* gene coupled with the suppression of the pancreatic secretion pathway might indicate an impaired ability to secrete digestive enzymes. Furthermore, digestive enzymes can catalyze the digestion and absorption of complex nutrients like proteins and fats [89]. Therefore, the observed downregulation of pathways (‘protein digestion and absorption’ and ‘fat digestion and absorption’) in exposed tadpoles revealed impairment of these essential metabolic functions. ‘Extracellular region’ and ‘extracellular space’ were the most significantly enriched categories identified in GO enrichment analysis of the BTr group and were also observed, though less prominently, in the ATr group. These two cellular component terms were also enriched in the hepatopancreas transcriptome of Asian clams (*Corbicula fluminea* Müller, 1774) exposed to MC-LR [90]. Plenty of downregulated genes related to these terms may reflect specific impairment of pancreatic exocrine function. In summary, the widespread downregulation of hepatic genes involved in digestion suggested a reduction in digestive function in Chinese brown frog tadpoles under MC-LR stress. Consequently, this may lead to nutrient malabsorption and gut microbiota dysbiosis. However, a limitation of this study is a failure to monitor the actual food intake of tadpoles in the different groups, which could account for differences in the nutritional status of the tadpoles observed under the various treatments.

The gut microbiota serves critical physiological functions in nutrient metabolism, immune regulation, and intestinal epithelial barrier maintenance [91,92,93], which could be affected by many factors, including diet, behavior, genetics, and environmental stress [94,95,96]. MC-LR has the capacity to enter and accumulate within intestinal epithelial cells, subsequently inducing gut microbiota dysbiosis by altering both taxonomic diversity and compositional profiles. Here, significant increases in Chao and Ace indices were found in the ATr group relative to the Con group. Both of them serve as nonparametric estimators of species richness [97]. The results demonstrated that the richness of the gut microbiota was significantly increased by MC-LR, which was consistent with the changes in alpha diversity indices seen in MC-LR-exposed zebrafish [98] and Pacific white shrimp [99]. In crayfish treated with MC-LR, however, the gut microbiota had reduced richness [22]. Additionally, the Chao and Ace indices in the BTr group decreased compared to those in the ATr group, but they were still higher than those in the Con group. A similar phenomenon was observed in young African clawed toads: the Chao and Shannon indices of the gut microbiota showed an initial rise, followed by a decrease, with increasing MC-LR concentration [38]. Although the variations in microbial alpha diversity caused by MC-LR remain controversial so far, it is still clear that MC-LR can alter gut microbial richness. Differences in species, exposure methods, and MC-LR dosage might contribute to the inconsistent findings across studies [38].

The gut microbiota composition was also impacted by MC-LR exposure, with changes in bacterial proportions. In Chinese brown frog tadpoles, the dominant phyla were Firmicutes, Desulfobacterota, Proteobacteria, and Fusobacteriota, which was consistent with changes seen in Asiatic toad (*Bufo gargarizans* Cantor, 1842) tadpoles [100]. A significant elevation in the proportion of Desulfobacterota and marked decreases in Fusobacteriota and Actinobacteriota were observed in the MC-LR-treated groups compared with the control group. Many studies have proposed that Desulfobacterota is correlated with the pathogenesis of inflammation and metabolic disorders [101]. Furthermore, Desulfobacterota can produce H_2_S, which would damage the integrity of intestinal barrier and promote the activation of inflammation [102]. In contrast, most members of the phylum Fusobacteriota are involved in the synthesis of vitamin B12 and vitamin B9 [103]. These bacteria also play key roles in the host, including suppressing immune cell cytotoxicity, recruiting inflammatory cytokines, and participating in inflammatory regulation [104,105]. Despite its relatively low abundance in the gut microbiota, Actinobacteriota plays an important role in maintaining intestinal homeostasis [106]. Members of this phylum are prolific producers of antibiotic secondary metabolites that defend against pathogenic invaders [107]. In addition, Actinobacteriota contributes to host health by decomposing complex carbohydrates and modulating immune responses [108,109]. Thus, an increase in Desulfobacterota, which has harmful effects, and decreases in Fusobacteriota and Actinobacteriota, which have beneficial effects, may increase disease susceptibility in the exposed tadpoles. A higher Firmicutes/Bacteroidetes ratio is associated with enhanced host capacity for energy absorption and storage [110]. In our study, however, the ratio was significantly reduced in both experimental groups, which may result from diminished energy harvesting efficiency under MC-LR stress [111,112]. This decrease was closely related to the observed downregulation of digestion in the hepatic transcriptome.

At the genus level, the ATr group showed a notably higher relative abundance of *Eubacterium* compared to the Con group. *Eubacterium* is a vital butyrate producer in the intestine [113], and studies have demonstrated that butyrate plays a critical role in anti-inflammation and maintaining gut barrier integrity [114]. The elevated abundance of *Eubacterium* may represent a compensatory response to intestinal damage induced by MC-LR exposure. However, the abundance of the genus *Pygmaiobacter* was decreased significantly at a concentration of 10.0 μg/L MC-LR. *Pygmaiobacter* is strongly associated with host health status and can promote the growth of beneficial intestinal bacteria and enhance the efficacy of the gut microbiome [115,116].

In brief, MC-LR exposure led to a significant increase in the relative abundance of harmful Desulfobacterota in tadpole intestines, while the abundances of several beneficial bacteria (e.g., Fusobacteriota, Actinobacteriota, and *Pygmaiobacter*) were markedly reduced. These alterations suggested that MC-LR may adversely affect both intestinal health and the homeostasis of the host by disrupting the gut microbiota structure.

The histopathological analyses confirmed significant structural alterations in the liver and intestinal tissues of MC-LR-exposed Chinese brown frog tadpoles. At the high MC-LR dose, the liver tissues exhibited notable damage characterized by necrosis and nuclear pyknosis and deformation. Consistent with our findings, MC-LR-induced histopathological damages were observed in the hepatopancreases of giant freshwater prawn (*Macrobrachium rosenbergii* De Man, 1879) [117] and crayfish [22]. Further, histological examination of the intestine revealed that histological damage was caused by MC-LR, such as pyknotic and deformed nuclei. Similarly, damaged intestinal structure was also found in MC-LR-exposed zebrafish [118] and Pacific white shrimp [30]. The observed histological alterations may result from oxidative stress caused by MC-LR in the tissues, which could cause cellular injury [60,119,120]. These findings suggested that MC-LR caused significant histological alterations in both the hepatic and intestinal tissues of tadpoles, thereby harming crucial physiological functions.

## 5. Conclusions

In conclusion, MC-LR exposure had severe toxic effects on the liver and intestine of Chinese brown frog tadpoles. The alterations in oxidative stress markers (MDA, SOD, GPx, and TAC) suggested that MC-LR could induce liver oxidative stress by enhancing lipid peroxidation and impairing the antioxidant defense system. Additionally, the liver cells exhibited downregulation of pathways related to digestion, providing molecular-level insights into functional disruption under MC-LR stress. Furthermore, bacterial alpha diversity indices (Chao and Ace) were increased in MC-LR-treated gut samples, and the relative abundances of dominant bacterial taxa were changed significantly. Histological damage was observed in both the hepatic and intestinal tissues of tadpoles following MC-LR exposure. All these results suggested that MC-LR exposure could harm the liver and intestine in anurans, highlighting a potential conservation threat to anurans in polluted freshwater ecosystems. Our work therefore provided important evidence to guide both ecological risk assessment and the development of species protection strategies.

## Figures and Tables

**Table 1:** Variations in microbial alpha diversity indices in Chinese brown frog tadpoles after MC-LR exposure.

Group	Chao	Ace	Shannon	Simpson
Con	396.91 ± 46.28 ^b^	416.78 ± 44.09 ^b^	3.40 ± 0.26 ^a^	0.11 ± 0.36 ^a^
ATr	554.41 ± 59.15 ^a^	581.91 ± 61.87 ^a^	3.49 ± 0.19 ^a^	0.10 ± 0.03 ^a^
BTr	496.27 ± 53.53 ^ab^	559.41 ± 114.91 ^ab^	3.52 ± 0.24 ^a^	0.10 ± 0.04 ^a^

Letters (a and b) indicate significant differences (*p* < 0.05) among the groups. Con: 0 μg/L MC-LR; ATr: 1.0 μg/L MC-LR; BTr: 10.0 μg/L MC-LR.

## Data Availability

The original contributions presented in this study are included in the article/Supplementary Material. Further inquiries can be directed to the corresponding author.

## Supplementary Materials

The following supporting information can be downloaded at: https://www.mdpi.com/article/10.3390/ani16020316/s1. Method S1: Library preparation and transcriptome sequencing; Method S2: PCR amplification; Method S3: Illumina Miseq sequence processing; Figure S1: Volcano plots of differentially expressed genes (DEGs) in the livers of Chinese brown frog tadpoles exposed to MC-LR; Figure S2: Heatmap showing the relationship between key differentially expressed genes (DEGs) and MC-LR exposure levels; Figure S3: Rarefaction curves of intestinal microbial samples from Chinese brown frog tadpoles, based on Illumina MiSeq sequencing; Figure S4: Principal coordinate analysis (PCoA) plots of data from intestinal microbial samples following MC-LR exposure for 7 days; Figure S5: Venn diagrams of OTU distribution; Table S1: Summary of transcriptome sequencing data from the livers of Chinese brown frog tadpoles; Table S2: Details of the top 20 enriched GO terms in the ATr group (1.0 μg/L MC-LR); Table S3: Details of the top 20 GO enriched terms in the BTr group (10.0 μg/L MC-LR); Table S4: Details of the significantly enriched KEGG pathways in the ATr group (1.0 μg/L MC-LR); Table S5: Details of the significantly enriched KEGG pathways in the BTr group (10.0 μg/L MC-LR); Table S6: Histopathological assessment of hepatic lesions in Chinese brown frog tadpoles following MC-LR exposure. References [121,122] are cited in the Supplementary Materials.
