# Supplementary material for: Microcystin-LR-Induced Oxidative Stress, Transcriptome Changes, Intestinal Microbiota, and Histopathology in Rana chensinensis Tadpoles
Source: Animals (Basel). 2026 Jan 20;16(2):316. doi: 10.3390/ani16020316 (PMC12837403; doi:10.3390/ani16020316)
Supplement: Supplementary file 1 [file animals-16-00316-s001.zip › animals-4012327-supplementary.pdf]

Supplementary Materials

Microcystin-LR-Induced Oxidative Stress, Transcriptome Changes, Intestinal Microbiota, and Histopathology in *Rana chensinensis* Tadpoles

You Wang[1], Bingjie Wang[1], Zhuolin He[1], Jiaxin Chen[1], Chenyang Liu[1], Zhanqi Wang[2], Muhammad Irfan[3] and Lixia Zhang[1, 4,*]

[1]*Department of Ecology, College of Life Sciences, Henan Normal University, Xinxiang 453007, China;*

[2]*Key Laboratory of Vector Biology and Pathogen Control of Zhejiang Province, College of Life Sciences, Huzhou University, Huzhou 313000, China;*

[3]*Department of Biotechnology, University of Sargodha, Sargodha 40100, Pakistan;*

[4]*Puyang Field Scientific Observation and Research Station for Yellow River Wetland Ecosystem, Puyang 457183, China*

**Correspondence: zhanglxsky@163.com*

1. Supplementary Methods

Method S1. Library preparation and transcriptome sequencing

Sequencing libraries were generated using the TruSeq RNA Sample Preparation Kit (Illumina, San Diego, CA, USA). Firstly, mRNA was purified from total RNA using poly-T oligo-attached magnetic beads. Fragmentation was carried out using divalent cations under elevated temperature in an Illumina proprietary fragmentation buffer. First strand cDNA was synthesized using random oligonucleotides and Super Script II. Second strand cDNA synthesis was subsequently performed using DNA Polymerase I and RNase H. Remaining overhangs were converted into blunt ends via exonuclease/polymerase activities and the enzymes were removed. After adenylation of the 3' ends of the DNA fragments, Illumina PE adapter oligonucleotides were ligated to prepare for hybridization. To select cDNA fragments of the preferred 400-500 bp in length, the library fragments were purified using the AMPure XP system (Beckman Coulter,Beverly, CA, USA). DNA fragments with ligated adaptor molecules on both ends were selectively enriched using Illumina PCR Primer Cocktail in a 15 cycle PCR reaction. Products were purified (AMPure XP system) and quantified using the Agilent high sensitivity DNA assay on a Bioanalyzer 2100 system (Agilent). The sequencing library was then sequenced on Illumina X PLUS platform (Illumina, USA) by Shanghai Personal Biotechnology Cp. Ltd.

Method S2. PCR amplification

The PCR was conducted in 20-μL reactions that contained 10 μL 2×Pro Taq, 0.8 μL upstream primer (5 μM), 0.8 μL downstream primer (5 μM), 10 ng template DNA. Thermocycling conditions included denaturation at 95 ℃ for 3 min, following by 29 cycles at 95 ℃ for 30 s, annealing at 53 ℃ for 30 s, extension at 72 ℃ for 45 s and final elongation at 72 ℃ for 10 min.

Method S3. Illumina Miseq sequence processing

The raw gene sequencing reads were de-multiplexed, quality-filtered, and merged using fastp (version 0.19.6) and FLASH (version 1.2.7) with the following criteria. Firstly, the reads were truncated at any site receiving an average quality score of < 20 over a 50 bp sliding window. The truncated reads shorter than 50 bp were

discarded, and reads containing ambiguous characters were also discarded. Secondly, only overlapping sequences longer than 10 bp were assembled according to their overlapped sequence. The maximum mismatch ratio of overlap region is 0.2. Reads that could not be assembled were discarded. Thirdly, samples were distinguished according to the barcode and primers, and the sequence direction was adjusted, with exact barcode matching and 2 nucleotide mismatches in primer matching. Then the optimized sequences were clustered into operational taxonomic units (OTUs) using UPARSE 7.1 with 97% sequence similarity level. The most abundant sequence for each OTU was selected as a representative sequence. The chloroplast and mitochondria sequences were removed from the data. The taxonomy of each OTU representative sequence was analyzed by RDP Classifier (version 2.2) against the 16S rRNA gene database (Silva v138) using confidence threshold of 0.7.

2. Supplemental Figures

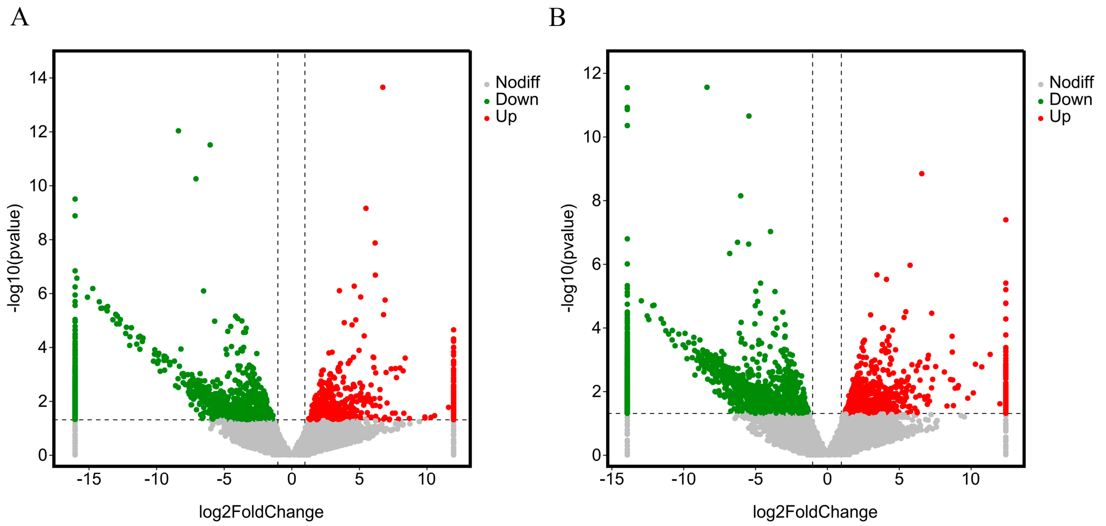

Figure S1. Volcano plots of differentially expressed genes (DEGs) in the livers of Chinese brown frog tadpoles exposed to MC-LR. (A) DEGs identified in 1.0 μg/L MC-LR-treated group compared to the control. (B) DEGs identified in 10.0 μg/L MC-LR-treated group compared to the control. Red dots represent up-regulated genes and green dots represent down-regulated genes.

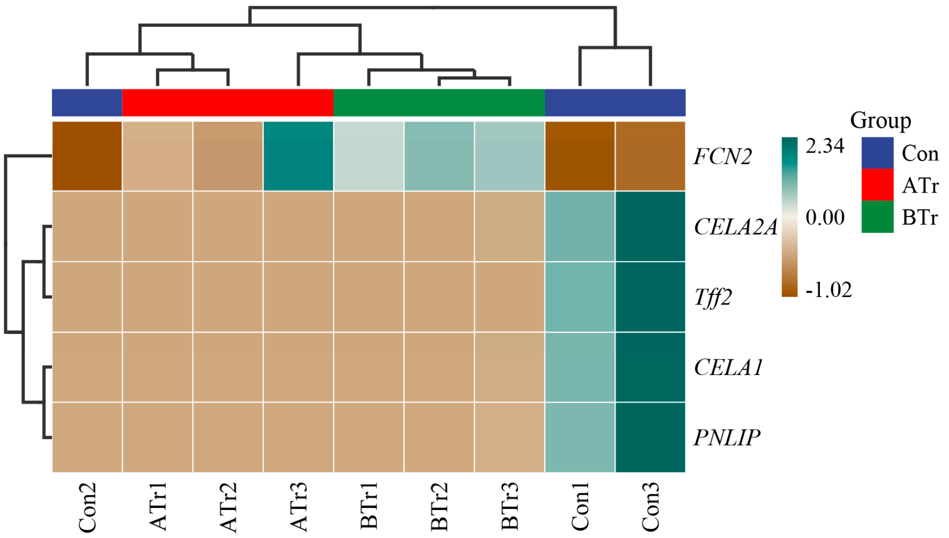

Figure S2. Heatmap showing the relationship between key differentially expressed genes (DEGs) and MC-LR exposure levels. Con: 0 μg/L MC-LR; ATr: 1.0 μg/L MC-LR; BTr: 10.0 μg/L MC-LR.

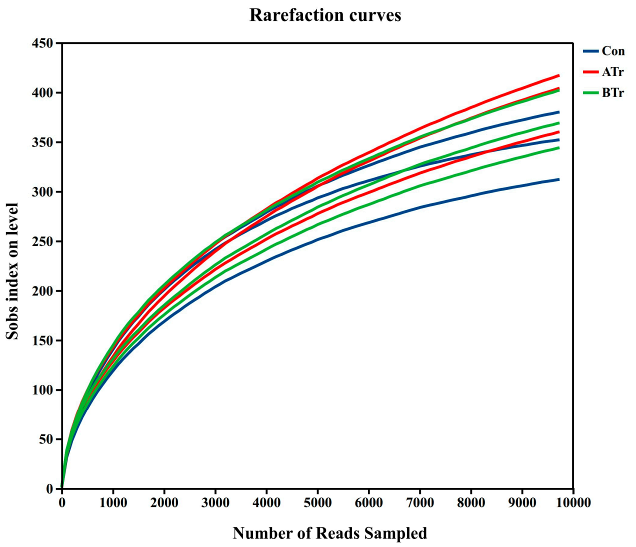

Figure S3. Rarefaction curves of intestinal microbial samples from Chinese brown frog tadpoles based on Illumina MiSeq sequencing. Con: 0 μg/L MC-LR; ATr: 1.0 μg/L MC-LR; BTr: 10.0 μg/L MC-LR.

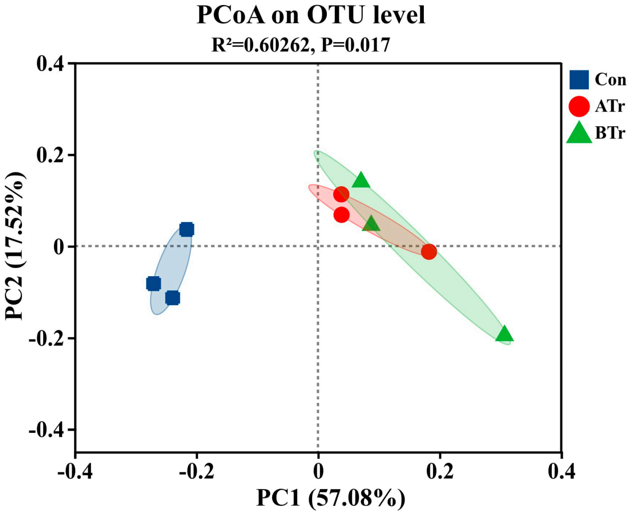

Figure S4. Principal coordinate analysis (PCoA) plots of data from intestinal microbial samples following MC-LR exposure for 7 days. Con: 0 μg/L MC-LR; ATr: 1.0 μg/L MC-LR; BTr: 10.0 μg/L MC-LR.

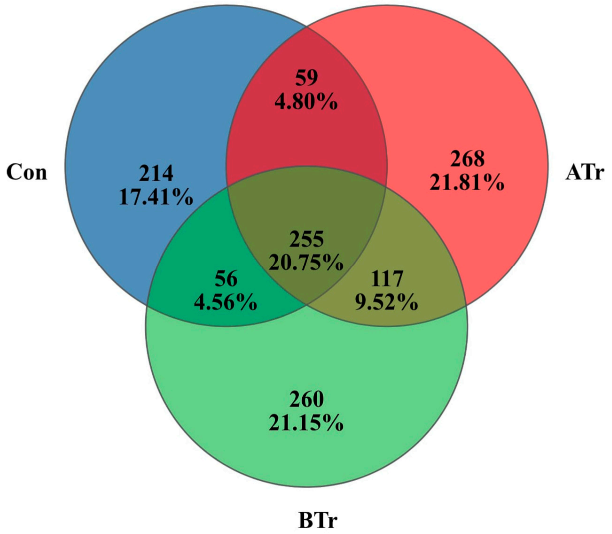

Figure S5. Venn diagrams of OTU distribution. The numbers and proportions of group-specific or shared OTUs are shown in the circles. Con: 0 μg/L MC-LR; ATr: 1.0 μg/L MC-LR; BTr: 10.0 μg/L MC-LR.

3. Supplementary Tables

Table S1. Summary of transcriptome sequencing data for the livers of Chinese brown frog tadpoles.

Sample	Raw reads	Raw bases (bp)	N (%)	Q20 (%)	Q30 (%)	Clean reads	Clean bases (bp)	Clean Reads (%)	Clean bases (%)
Con1	51766580	7816753580	0.007021	98.76	96.72	50907326	7670797776	98.34	98.13
Con2	53858774	8132674874	0.006908	98.89	97.08	53035394	7990664526	98.47	98.25
Con3	48296124	7292714724	0.006950	98.88	97.01	47595232	7172035590	98.55	98.35
ATr1	53512318	8080360018	0.006854	98.70	96.61	52563472	7913624166	98.23	97.94
ATr2	49115240	7416401240	0.007493	98.67	96.54	48215632	7262034776	98.17	97.92
ATr3	54922820	8293345820	0.006956	98.88	96.98	54115970	8155046175	98.53	98.33
BTr1	55040384	8311097984	0.007119	98.78	96.76	54152640	8159220557	98.39	98.17
BTr2	51248458	7738517158	0.007068	98.74	96.66	50370880	7588940962	98.29	98.07
BTr3	48638920	7344476920	0.006987	98.77	96.70	47832576	7208901329	98.34	98.15

Con: 0 μg/L MC-LR; ATr: 1.0 μg/L MC-LR; BTr: 10.0 μg/L MC-LR.

Table S2. The details of top 20 enriched GO terms in ATr group (1.0 μg/L MC-LR).

Category	GO.ID	Term	Up	Down	DEG	FDR
BP	GO:0007586	digestion	1	71	72	7.27346E-47
MF	GO:0004252	serine-type endopeptidase activity	7	61	68	1.96404E-43
MF	GO:0008236	serine-type peptidase activity	8	61	69	6.10689E-41
MF	GO:0016825	hydrolase activity, acting on acid phosphorus-nitrogen bonds	8	61	69	1.67597E-40
MF	GO:0017171	serine hydrolase activity	8	61	69	1.67597E-40
CC	GO:0005615	extracellular space	29	117	146	8.09035E-38
CC	GO:0005576	extracellular region	50	154	204	7.28573E-33
CC	GO:0097180	serine protease inhibitor complex	0	24	24	1.47574E-29
MF	GO:0004175	endopeptidase activity	11	68	79	3.55352E-29
MF	GO:0070011	peptidase activity, acting on L-amino acid peptides	12	81	93	4.79014E-29
MF	GO:0008233	peptidase activity	13	82	95	4.79014E-29
MF	GO:0097655	serpin family protein binding	0	22	22	8.08474E-28
CC	GO:0097179	protease inhibitor complex	0	24	24	8.31422E-24
CC	GO:1904090	peptidase inhibitor complex	0	24	24	8.31422E-24
BP	GO:0009235	cobalamin metabolic process	1	24	25	5.28514E-19
BP	GO:0060309	elastin catabolic process	0	14	14	3.0291E-16
BP	GO:0061113	pancreas morphogenesis	0	13	13	6.60611E-15
BP	GO:0051541	elastin metabolic process	0	14	14	9.561E-15
CC	GO:0031514	motile cilium	0	39	39	1.15386E-15
CC	GO:0060102	collagen and cuticulin-based cuticle extracellular matrix	1	12	13	2.29603E-15

Table S3. The details of top 20 GO enriched terms in BTr group (10.0 μg/L MC-LR).

Category	GO.ID	Term	Up	Down	DEG	FDR
CC	GO:0005576	extracellular region	92	252	344	1.92232E-59
BP	GO:0007586	digestion	1	91	92	5.9523E-52
CC	GO:0005615	extracellular space	55	165	220	1.82388E-51
MF	GO:0004252	serine-type endopeptidase activity	10	65	75	1.50793E-35
MF	GO:0008236	serine-type peptidase activity	12	65	77	1.59386E-33
MF	GO:0016825	hydrolase activity, acting on acid phosphorus-nitrogen bonds	12	65	77	4.92744E-33
MF	GO:0017171	serine hydrolase activity	12	65	77	4.92744E-33
MF	GO:0008233	peptidase activity	20	109	129	1.43161E-30
MF	GO:0070011	peptidase activity, acting on L-amino acid peptides	20	105	125	4.41564E-30
MF	GO:0004175	endopeptidase activity	17	85	102	7.86411E-29
CC	GO:0097180	serine protease inhibitor complex	0	24	24	3.61609E-24
MF	GO:0097655	serpin family protein binding	0	22	22	8.48058E-23
CC	GO:0097179	protease inhibitor complex	0	24	24	1.5825E-18
CC	GO:1904090	peptidase inhibitor complex	0	24	24	1.5825E-18
BP	GO:0009235	cobalamin metabolic process	0	26	26	8.27944E-15
BP	GO:0006952	defense response	101	105	206	2.01389E-14
BP	GO:0007584	response to nutrient	16	55	71	2.04803E-12
BP	GO:0022600	digestive system process	1	37	38	2.12571E-12
MF	GO:0016787	hydrolase activity	48	205	253	3.9395E-13
BP	GO:0061113	pancreas morphogenesis	0	13	13	3.91298E-12

Table S4. The details of significantly enriched KEGG pathways in ATr group (1.0 μg/L MC-LR).

Pathway ID	Pathway	Level2	Up	Down	DEG	FDR
ko04972	Pancreatic secretion	Digestive system	0	50	50	2.77803E-36
ko04974	Protein digestion and absorption	Digestive system	0	42	42	6.31302E-31
ko04080	Neuroactive ligand-receptor interaction	Signaling molecules and interaction	7	27	34	4.75936E-13
ko04975	Fat digestion and absorption	Digestive system	0	7	7	0.010296765
ko04970	Salivary secretion	Digestive system	1	9	10	0.019108403

Table S5. The details of significantly enriched KEGG pathways in BTr group (10.0 μg/L MC-LR).

Pathway ID	Pathway	Level2	Up	Down	DEG	FDR
ko04974	Protein digestion and absorption	Digestive system	0	50	50	1.47491E-25
ko04972	Pancreatic secretion	Digestive system	0	52	52	4.62368E-23
ko04080	Neuroactive ligand-receptor interaction	Signaling molecules and interaction	9	35	44	7.33349E-10
ko04975	Fat digestion and absorption	Digestive system	1	13	14	5.28024E-06
ko04977	Vitamin digestion and absorption	Digestive system	1	11	12	0.000848704
ko00980	Metabolism of xenobiotics by cytochrome P450	Xenobiotics biodegradation and metabolism	1	10	11	0.021719297
ko04613	Neutrophil extracellular trap formation	Immune system	17	6	23	0.021748081
ko04145	Phagosome	Transport and catabolism	20	8	28	0.021748081
ko00982	Drug metabolism - cytochrome P450	Xenobiotics biodegradation and metabolism	0	11	11	0.021748081
ko03320	PPAR signaling pathway	Endocrine system	0	15	15	0.024431374
ko04979	Cholesterol metabolism	Digestive system	1	9	10	0.049886113

Table S6. Histopathological assessment for hepatic lesions observed in Chinese brown frog tadpoles following MC-LR exposure.

Score	Histological characteristics
0	Normal hepatic structure
1	Normal hepatic structure
	Mild fatty vacuolation of hepatocytes
2	Normal hepatic structure
	Moderate fatty vacuolation of hepatocytes
	Mild hyperemia of hepatic blood vessels
3	Typical hepatic structure + 2–5 of the following
	Severe fatty vacuolation of hepatocytes
	Moderate to severe hyperemia of hepatic blood vessels
	Hepatocellular hyperplasia
	Hepatocellular hypertrophy
	Nuclear alterations
	Hepatocellular necrosis
	Hepatocellular fibrosis
	Other damage
4	Loss of hepatic structure with 2–5 of the histopathological alterations listed in score 3

The histopathological scoring system was adapted from Pierce et al. (1978) [1] and Bernet et al. (1999) [2]. 0–2: considered normal; 3–4: considered pathological.

References

1. Pierce, K.V.; McCain, B.B.; Wellings, S.R. Pathology of hepatomas and other liver abnormalities in English sole (*Parophrys vetulus*) from the Duwamish River estuary, Seattle, Washington. *J. Natl. Cancer Inst.* **1978**, *60*, 1445-1453.
2. Bernet, D.; Schmidt, H.; Meier, W.; Burkhardt-Holm, P.; Wahli, T. Histopathology in fish: proposal for a protocol to assess aquatic pollution. *Journal of fish diseases* **1999**, *22*, 25-34.
